# The Slow Flower Movement – exploring alternative sustainable cut-flower production in a Swedish context

**DOI:** 10.1016/j.heliyon.2022.e11086

**Published:** 2022-10-13

**Authors:** Rebecca Thörning, Åsa Klintborg Ahlklo, Sara Spendrup

**Affiliations:** a Department of People and Society, Swedish University of Agricultural Sciences, PO Box 190, 234 22, Lomma, Sweden; b Department of Landscape Architecture, Planning and Management, Swedish University of Agricultural Sciences, PO Box 190, 234 22 Lomma, Sweden

**Keywords:** Slow flower movement, Cut-flower, Triple layered business model canvas (TLBMC), Sustainability

## Abstract

This study critically analyses the emerging Slow Flower Movement in a Swedish context. Analysis is conducted by using the Triple-layered Business Model Canvas, covering economic, social and environmental sustainability values. Data are collected (Spring 2021) through semi-structured interviews, observations of cultivation and use of social media. Analysis and coding were conducted by applying thematic analysis. The results show that consumer demand and market for domestic cut-flowers are increasing; however, the development is hindered by small scale production, low profitability and demanding work conditions. Results also show that produce, in line with the Slow Flower Movement, provides unique sustainable values: contribution to biodiversity, a different and unique assortment, functions as a meeting point, and contributes to the local culture, landscape and society. Social media represent a crucial tool in sales, marketing and communication, as well as in the overall development of the movement. Highlighting how these technological communication platforms constitute a foundation for the movement's establishment. The avoidance of technical production aid in the cultivation is regarded as a main hindrance to developing the firms and profitability. By initiating knowledge exchange between conventional growers and the emerging Slow Flower Movement, both parties are expected to gain advantages of a transformation towards a mutual development of sustainable domestic cut-flower production.

## 1 Introduction

Currently, an emerging and internationally growing movement called the Slow Flower Movement (Prinzing, 2020) is gaining momentum, presenting itself as a sustainable alternative to the conventional cut-flower industry. The main goal(s) of this movement is to preserve domestic flower farms and support a floral industry that builds on local, seasonal, sustainable, and safely farmed flowers and foliage (Slow Flower Society, 2021). The movement originates from northern America and has a clear ideological relationship with the agenda of the Slow Food Movement (Prinzing, 2013b). It claims to act as a social and political movement, working against the dehumanising effects of the large-scale commercial food industry (The Slow Movement, 2021), linked to the term of “*Slow Gardening*”, coined by the author and gardener Felder Rushing (Prinzing, 2013a). Prinzing (2013b) formulated the expression “Slow Flower” after reading Stewart (2008), criticising the conventional flower industry. The ideological connection to the Slow Food Movement forms an explicitly central part of the “Slow Flower” expression stated by Prinzing (2013b), which, in turn, represents the starting point of the Slow Flower Movement in the US in 2013 (Slow Flower Society, 2021).

*Thanks to the culinary pioneers who popularized the Slow Food movement, it now seems like you can put “slow” in front of any term to convey a different philosophy or approach to that subject. When I say the phrase “slow flowers,” there are those who immediately understand it to mean: I have made a conscious choice.*Prinzing (2013b, p.12).

Movements linked to the idea of slowness have often been found to build on a dichotomy, suggesting that fast is bad and slow is good, and where the latter is in line with a good and ethical life, mental well-being and democracy (Vostal, 2019). Critical voices questioning the agenda of the Slow Movement elaborate on, e.g. the fact that products and services mainly appear to be relevant for privileged consumer groups (middle class metropolitan dwellers), at times build on a rhetoric that underpins a localist and traditional mindset, may be closely linked to cultural and political tendencies touching on nationalism, and finally, build on the idea of authenticity and an emotional conviction that the external (fast world) is a threat (Vostal, 2019). The link between the Slow Flower Movement and the Slow Food Movement is thus an important condition to consider when exploring the phenomenon of the Slow Flower Movement. Today's Swedish domestic production of field-grown cut-flowers, produced in line with the Slow Flower Movement, represents a niche within the conventional cut-flower market.

### 1.1 The global floriculture market

Most of all the cut-flowers sold are mainly produced within the dominating, conventional floriculture industry, representing a high value global industry and supply chain, covering commercial production of cut-flowers and greens, breeding plants, cuttings, seeds and bulbs (Sharma, 2019; Rikken, 2010). The global market for flowers and ornamental plants was worth 42,4 billion USD in 2019 (Petal Republic, 2022). Europe, China and USA represent the dominant markets (Eurostat, 2020; Darras, 2020), with the Netherlands operating as the centre of the world's largest trading system for cut-flowers and plants (Petal Republic, 2021). The supply of cut-flowers, floriculture, is highly dependent on fast international transports (airborne transportation) as well as energy intensive high-tech production methods (irrigation, breeding, planting, spraying, harvesting, processing, refrigerating, packing and distributing (Gebhardt, 2015)). Lately, a substantial amount of floriculture production has been relocated to developing countries, mainly due to more favourable climate and growing conditions, less expensive labour costs and export/import policies (Ahmad et al., 2020). Consumption of cut-flowers is, on the other hand, still highest in the EU, USA and Japan (Xia et al., 2006), implying e.g. the European (and Swedish (Sundh, 2016)) market's dependency on import from distant countries such as Ecuador, Colombia, Israel, USA, Costa Rica, China and Uganda. Lately, the environmental impact (use of chemicals, water, heating of greenhouse), working environmental conditions (Hughes, 2001; Rikken, 2010; Stoessel et al., 2012), high dependency on air-cargo supply chain (due to short life of cut flowers) (Button, 2020) and “flower miles” (Manikas et al., 2020) have received attention. This has created a debate, questioning sustainability aspects within the conventional floriculture production system (Darras, 2020; Hughes, 2001; Rikken, 2010; Stoessel et al., 2012; Button, 2020; Manikas et al., 2020). As a response the industry has, for example, developed and implemented social and environmental standards, covering business-to-business standards but also labels directed towards consumers, such as Fair trade (Rikken, 2010; Ntinas et al., 2020) and the Flower label Programme (Rikken, 2010). Several measures have also been identified, with the aim of reducing the floricultural industry's climate impacts. One example addresses the mitigation of greenhouse gas (GHG) when using green houses, especially in the northern hemisphere, suggesting alternative renewable energy sources (Ntinas et al., 2020).

Identified negative consequences that exist in today's conventional production clarify the importance of finding solutions for a future sustainable production (floriculture). However, delivering value and reaching profitability are central to whether emerging cut-flower, field grower, companies may be profitable enough to develop and contribute to such a sustainable production system. Following this, the aim of this study is to critically analyse the Slow Flower Movement in a Swedish context. Specifically, we aim to critically explore how Swedish cut-flower field growers develop their business models and strategies to create value (economic, environmental and social) for their customers and enable income for themselves. Finally, the study addresses the ideological link to the slowdown movement and how this link strengthens or impedes the development of these firms.

The Slow Flower Movement is, fairly unexplored in a scientific context, not the least from a Swedish perspective. The main contribution of this exploratory study is the analyses of the phenomenon in a scientific critical context, by using a lens of sustainability.

### 1.2 Swedish floriculture

From a Swedish perspective, it has been suggested that the Nobel Prize banquet in 2018 represents the starting point for the Slow Flower Movement's existence in Sweden. The theme of the 2018 festivities comprised Swedish locally produced food and upcycled dresses/clothing. Since the origin of the cut-flowers was not considered in the practical implementation of the theme, the credibility of the sustainability theme was questioned (Björk, 2019). The debate that followed laid the foundation for the movement in Sweden (Snittblomsodlare i Sverige, 2020), which, in turn, has been believed to be a main driver for an increased general interest in domestically produced cut-flowers (Sveriges Radio, 2021).

Even though Sweden, today, imports most of the cut-flowers sold, this has not always been the case. On the contrary, historically, Sweden used to produce a great diversity and volume of cut-flowers. However, due to international competition and low transportation costs, many of those firms have either closed or changed production focus to e.g. vegetables (Sjöberg, 1949; Järna, 2017). A brief historic review shows that production in greenhouses expanded during the early 1900s, mainly driven by an increased demand and flower shops growing in popularity (Olausson, 2014). During the First World War, closed borders gave domestic growers exclusive rights to the market, which favoured the development of growers, a development that continued even after the war (Olausson, 2014). During the 1930s, many Swedish growers remained small producers of cut-flowers, often following a strategy of specialisation. At this time, Swedish domestic cultivation lasted all year, yet was supplemented by import during the winter months (Ekman, 1934). Technical development during the 1940s was a real game-changer for the Swedish industry, particularly for the greenhouse producers (Örtbrant, 2006). During the Second World War, the Netherlands cut-flower production became more industrialised (Gebhardt, 2015). Örtbrant (2006) explains that the Swedish production was weighed down by high costs, which made it difficult to compete with producers in countries with more favourable climate conditions. This, in combination with low transport costs, made it difficult for Swedish growers to compete with southern European growers. The decline in Swedish cut-flower producers continued throughout the years (Järna, 2017). Still, even though the Swedish cut-flower production is not as comprehensive and diversified as it once was, domestic conventional growers do exist. Today's Swedish cut-flower production is dominated by greenhouse tulip production, constituting 95% of the present Swedish flower production, which account for a production value of SEK 330 million (Jordbruksverket, 2019). In addition to this, several small-scale growers, both connected to the Slow Flower Movement (Snittblomsodlare i Sverige, 2020) and unorganised actors, have emerged during the last few years. Today the number of companies that are affiliated with the cut-flower growers' association has increased from around 30 members in 2020 to over 100 members in 2021 (Jordbruksaktuellt, 2021).

## 2 Theoretical framework – the business model concept and the value proposition

The business model and the value proposition that a firm applies and delivers is key in developing profitable and resilient firms. Generally, a business model is described as a firm's plan for creating and delivering value to customers, as well as describing the configuration of revenues and costs (Teece, 2010). Boons and Lüdeke-Freund (2013) explain how the concept is structured in accordance with (1) value proposition, (2) supply chain, (3) customer interface and (4) financial model and thus, may function as a tool to evaluate a firm and handle strategic and innovative work (Magretta, 2002). The value delivered by firms operating in the conventional cut-flower industry is heavily relying on a global value chain, economies of scale, optimization, and specialization (Carton and Parigot, 2021). The authors further argue that locally grown cut-flowers can improve industry sustainability through shifting from exploitation of endangered natural resources to a system that instead relies on local resources. The authors also explain that such a shift implies a rethinking in how and what value is created and offered to the consumer as well as the development and implementation of new value chains. Firms are suggested to focus on a value building on what is described as alternative natural resources' intrinsic properties. From a practical perspective such a strategy could be to choose varieties that are adapted to the local climate which in turn increases the vase-life of the cut-flowers and sale of untraditional varieties, compared to the conventional assortment.

The study has a focus on the value delivered to the consumers and how the growers create and communicate the value. A distinctive feature for many cut-flower growers is that they are active on social media, e. g Instagram, suggesting that these tools are of high importance in delivering value and implementing their business model. The importance of social media among small businesses has been explored by Jones et al. (2015). Identified benefits are related to increased awareness as well as inquiries, building relationships with customers and the actual number of (new) customers and finally support the co-promotion of other local businesses. It is further argued that by delivering meaningful content the firm may gain in awareness and revenues. The Business Model Canvas (BMC), originally developed by Osterwalder (2004), provides one relevant tool to use when exploring business models and value delivered. The BMC captures different elements within a business and facilitates observation, creating scenarios and future paths to pursue (Osterwalder, 2004). Since the introduction of the BMC, different improved versions of the original BMC (Osterwalder, 2004) have been suggested. In this study, we apply the Triple-Layered Business Model Canvas (TLBMC) by Joyce and Paquin (2016), which adds two layers to the economic aspect: the social and environmental. By using all three layers, the model supports a broader system of thinking and interconnections across different values, when studying a company's business model. Following this, the TLBMC is suggested to support a more robust and holistic view of the examined business model (Joyce and Paquin, 2016).

Within this study, the TLBMC is applied to structure the analysis of the business model and simultaneously enable an analysis of sustainability issues related to the Slow Flower Movement. Thus, the model supports a triple bottom line approach, addressing integrating and interlinking value creation concerning the economic, environmental, and social value (Joyce and Paquin, 2016).

## 3 Materials and method

**Figure 1:**
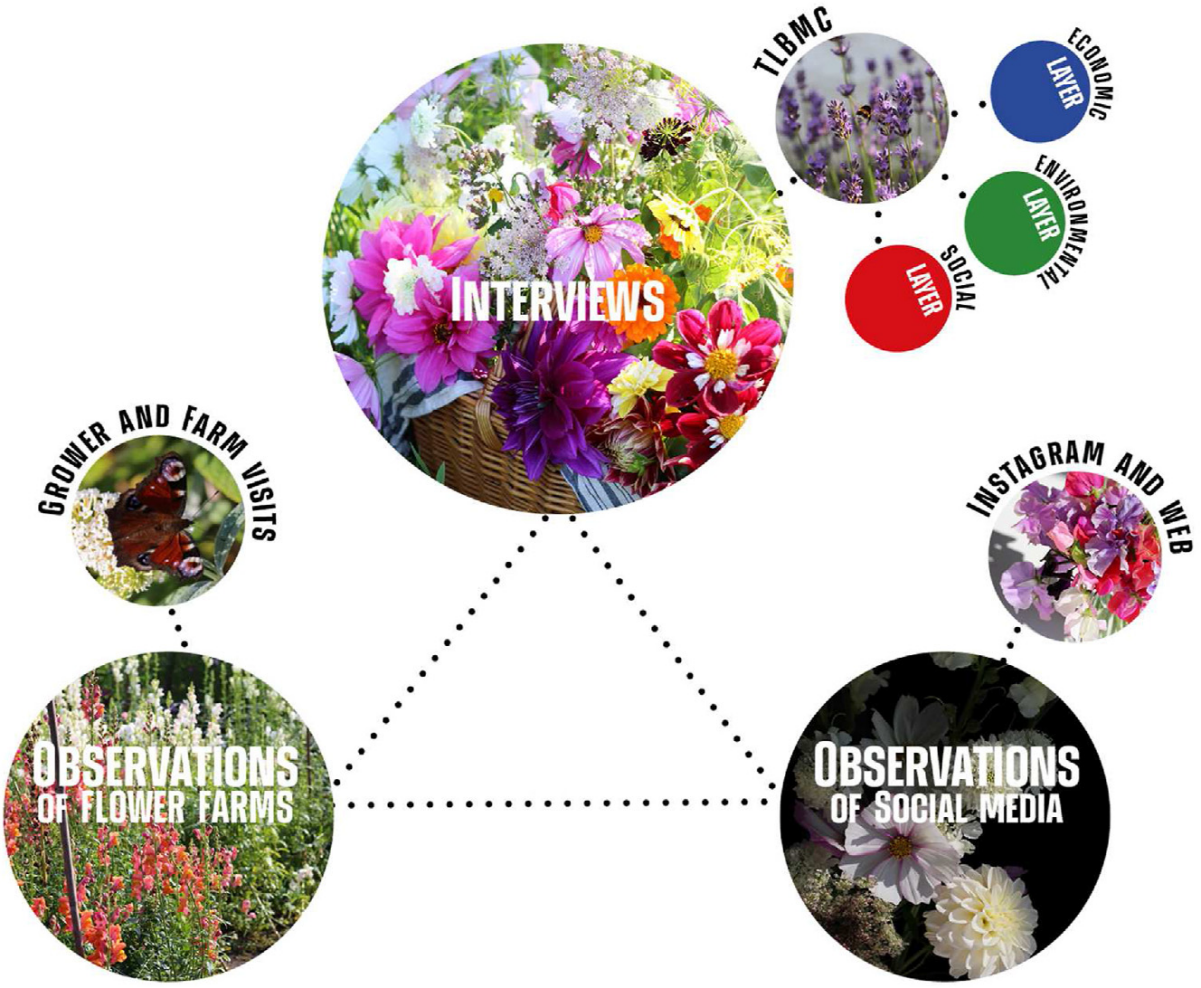
Description of method and linkage to theoretical frameworks applied in the analyses.

The presented study investigates a contemporary and developing phenomenon – the Swedish Slow Flower Movement. The scientific approach in implementing the present study interlinks a positivistic and an interpretivist approach. Positivistic since the study evaluates and present the result in relation to a structured protocol (TLBMC). Interpretivism, considering how the study has a special focus on the growers and how the result is retrieved through the lens of the grower's reality. Based on these conditions, a qualitative methodological approach was deemed most appropriate, combining information sourced through: (1) interviews with cut-flower field growers, (2) observations on the cut-flower field growers' farms and (3) observations of growers applied social media use (See Figure 1). By combining these three information sources and reach triangulation we expected to reach a greater depth in the retrieved data (Patel and Davidson, 2011; Yin, 2018).

Implementation of the study followed the Swedish University of Agricultural Sciences policy for processing personal data (https://www.slu.se/en/about-slu/contact-slu/personal-data/). Participation was voluntary, and information was provided about content and purpose. A signed consent form was obtained from all interviewees prior to the interview. Data were coded, ensuring anonymity. National and international agreements were followed. The general international code and guidelines on market and social research used by the International Chamber of Commerce (ICC/ESOMAR, 2016) were followed.

### 3.1 Selection of growers

The selection of growers was based on two criteria: (1) the grower had to have a registered firm and grow and produce field-grown cut-flowers for commercial sales and (2) the selection was based on respondents who could both participate in a personal interview and provide a physical presentation of the actual growing unit. Respondents were recruited through convenience sampling (Bryman, 2011), i.e. through the community Cut Flower Growers in Sweden (=Snittblomsodlare i Sverige) and Ising and Koller (2021). For practical reasons the geographic location of the interviewees was limited to the southern part of Sweden. An overview of the included cases is presented in Table 1.

**Table 1:** Background information of the cut-flower field growers and production sites.

	Age	Gender	Education and profession	Start date of firm	Cultivation area (m^2^)	Member of “Cut Flower Growers in Sweden”	Main income from cultivation
R1	39	Female	Biologist (former profession)	2020	500	Yes	Yes (complementing with extra job)
R2	57	Female	Landscape architect and university teacher (present main occupation)	2020	400	No	No
R3	52	Female	Nurse (former profession)/gardener	2020	100	Yes	Half-time (+half-time as hired gardener)
R4	47	Female	Real estate dealer/economist (former profession)	2018	1000	Yes	Yes (complementing as interior consultant)

### 3.2 Data collection

Semi-structured interviews were conducted with growers using an interview guide. To cover data both related to included growers, as well as contextual data questions included in the interview guide cover sociodemographic information of participating growers, production-specific questions of growing cut-flowers following a Slow-Flower approach, growers' applied social media communication channels, as well as topics covered in the Triple-Layered Business Model Canvas (TLBMC) (Joyce and Paquin, 2016): economic, environmental and social aspects related to the respondents’ business models when growing cut-flowers (see Table 2).

**Table 2:** Included themes in the interview guide, covering the economic, environmental, and social dimensions of the TLBMC by Joyce and Paquin (2016).

Economic	Environmental life cycle	Social stakeholder
Partners	Supplies and out-sourcing	Local communities
Activities	Production	Governance
Resources	Materials	Employees
Value proposition	Functional value	Social value
Customer relationship	End-of-life	Societal culture
Channels	Distribution	Scale of outreach
Customer segments	Use phase	End-user
Costs	Environmental impacts	Social impacts
Revenues	Environmental benefits	Social benefits

Interviews (R1–4, see Table 1) were conducted through Zoom (Zoom Video Communications Inc., 2022), audio- and video-recorded, conducted between March/April 2021 and lasted between 90 and 120 min.

The farm visits were formed as unstructured observations (Patel and Davidson, 2011), carried out between March/April 2021. The aim of the observations was to get a deeper understanding of the work conducted in the cultivation and to provide the interviewer with an opportunity to seek clarification as well as follow-up questions in relation to the interviews already conducted on zoom. Respondents presented their cut-flower field in its current state (off season). Notes and photos (with consent from the respondents) were taken to document the observations. The farm visits thus mainly added a practical context and framework for the information retrieved during the interview, as well as an understanding of the unique qualities of the place.

Observation of use of social media was additionally applied following an unstructured observations methodology (Patel and Davidson, 2011) and social media (e.g. Instagram and web pages) of all participating growers was visited. The aim of these observations was to get a richer picture and understanding of the growers' applied digital communication, interaction with customers and use of social media.

Considering the three different data sources (interviews in combination with observations of farms as well as use of social media), it is important to clarify that the interviews form the basis for the analysis and that observations of farms and social media are complementary (see Figure 1).

### 3.3 Data analysis

The audio recordings of the interviews were transcribed by using the programme oTranscribe (oTranscribe, 2021). The analysis and coding were conducted by applying thematic analysis (Boyatzis, 1998), implemented through the process of sorting, reducing, and analysing (Rennstam and Wästerfors, 2015). Initially the transcribed interviews were systematically evaluated, and statements were coded into the three predefined themes constituting the Triple-Layered Business Model Canvas (TLBMC) (Joyce and Paquin, 2016), see Table 2. In addition to this, sociodemographic data and production specific matters were coded into a separate theme. Secondly data for each theme was collected into four documents (1. Economic, 2. Environmental life cycle, 3. Social stakeholder and 4. Sociodemographic data and production). The material (document 1–3) was then analysed and coded in line with the subthemes of the three different layers (see Table 2) and clustered in line with these. The material was organised to illustrate differences as well as similarities between the interviewees, when analysed through the lens of the TLBMC. Sociodemographic data and production data was summarised in table format, see Table 1 and described in 4.1. Thirdly, findings from the farm visits (notes and photos) and social media observations (Instagram and web pages) were scrutinized in order to correlate and confirm the result obtained from the interviews. The aim of the triangulation was to ensure validity and verify the outcome of the primary data source, the interviews.

## 4 Results and discussion

The following section is structured with an initial description of the growers (see Table 1 for additional details (4.1)), followed by an analysis of the triple layered business model (4.2–4.4). Summaries of main findings of the three layers of the TLBMC are presented in Table 3 (economic), Table 4 (environmental) and Table 5 (social).

**Table 3:** Summary of main findings of elements within the economic layer of the TLBMC.

Economic	Main findings
Partners	National knowledge network among Slow Flower growers in Sweden, membership in production communities and local (female) entrepreneurial networks, international contacts
Activities	Cultivation, marketing, sales, customer relations (social media)
Resources	Access to land and water, family (work contribution), technical equipment and production input (allowed), seeds, muscle strength, skills in social media and photography
Value proposition	Flowers produced by the use of craftmanship, ban of pesticides and mineral fertilisers, no long-distance transports. Production supporting local economy. An alternative to conventional production, building on a different assortment and uniqueness in quality (wilderness and imperfection). Co-creation between growers and customers
Customer relationship	Providing flowers for decoration, gifts, weddings, funerals, etc. The communication is a major part of the business model, either through direct communication and physical meeting or through social media
Channels	Self-picking, flower trolleys, stores and local farm shops, short food supply chains, home delivery, event planners, restaurants, florists, flexible and generous opening-hours.
Customer segment	Mainly women, age 25 and older, large geographic spread in customers' domicile
Costs	High in start-up phase due to investments in cultivation, LED-lights and supply for production. Labour costs dominating when established. Difficulties with profitability
Revenues	Sales of flowers, courses, lectures and sales of refreshments

**Table 4:** Summary of main findings of elements within the environmental layer of the TLBMC.

Environmental	Main findings
Supplies and outsourcing	Local sales channels, avoidance of long-distance when selling. Relying on import for seeds and bulbs
Production	Manual work, avoidance of fossil fuel, pesticides, mineral fertilizers. Focus on local production and consumption
Materials	Depending on purchase of production material and the industry that provides the platform(s) for social media. Making conscious variety choices in order to select varieties that are robust and have a long shelf-life
Functional value	The average weight of different varieties bought in the span of one year, by one customer
End-of-life	The product is compostable, and growers use compostable material (e.g. thread applied), thus reducing waste and supporting circularity
Distribution	Car, bike, by foot and public transportation.
Use Phase	Short time lapse between harvest and consumption, leading to long durability (vase life)
Environmental Impact	Transportation of production material, customers travelling to the location
Environmental Benefits	Contributing to biodiversity

**Table 5:** Summary of the main findings of elements within the social layer of the TLBMC.

Social	Main findings
Local communities	Part of local business networks
Governance	Growers are one-woman entrepreneurs, no employees, often dependent on family members for unpaid work contribution
Grower (Employees)	The growers are central to developing the strong relationship with the customers. Value is co-created between the growers and customers by providing knowledge of techniques and aesthetic aspects to consider when customers produce their own bouquets. Appreciate working in the field mainly due to the perceived beauty of the plants and the surroundings
Social value	Functions as an event and social gathering, contributing to the local culture and society
Societal culture	Informing customers about environmental considerations and benefits of the products
Scale of outreach	Relations with other growers and other local entrepreneurs. Developing strong relations with customers (both in person and through social media)
End-user	The customers buying the flowers or looking for a trip/cultural event and a place to socialise
Social impacts	Long working hours, stressful to administrate communication and at times two employees, allowing customers to dwell in one's private garden, flexibility – never time off
Social benefits	Add aesthetic qualities to the area and location, both for growers, customers, neighbours and people passing by

### 4.1 The growers

All growers learned about the Slow Flower Movement through social media or magazines; moreover, they are female and started their businesses between 2018 and 2020. They all provided different reasons for starting their businesses; however, a recurrent theme was described as a need to radically change their way of living and/or to develop their hobby of growing flowers into a business opportunity. All growers are in rural areas in the southern part of Sweden, and the size of the production units varies from 100 m^2^ to 1000 m^2^. A majority of the growers do not have a traditional horticultural educational background or work experience; instead, they mainly come from completely different industries (see Table 1 for details).

### 4.2 The economic layer

The work tasks and activities that the growers carry out in their companies are mainly related to cultivating and marketing. Additional activities relate to networking, identifying new resellers and potential buyers in the surrounding areas, as well as arranging workshops directed towards consumers. The growing season is limited by weather conditions, which means that the season normally begins in March/April and may continue until October/November. During high season (April–October), time is mainly spent on cultivation and marketing. During the low season (November–February), growers evaluate the previous year and plan next year's production, e.g. buying seeds, tubers and bulbs. Seeds are sown during late winter and/or early spring. The typical customer is a woman, age of 25 and up.

As previously described, the Swedish domestic production of cut-flowers has been declining during recent years (Örtbrant, 2006), which could be assumed to have resulted in a lack of knowledge transfer between established growers and new entrants. This is, however, not mentioned as a problem to the new growers. Instead, the growers rely on expertise and knowledge exchange within the networks that they are part of in the association Cut Flower Growers in Sweden and do not use traditional horticultural advisory organisations for counselling. Several growers are members of the cut-flower growers’ association in Sweden and follow the specific requirements for production: to preserve domestic “*flower farms*” and support a floral industry that builds on local, seasonal, sustainable, and safely farmed flowers and foliage (Snittblomsodlare i Sverige, 2020). However, not all growers are members; as an example, one grower has made the deliberate choice not to join the community of cut-flower growers in Sweden, mainly due to already established networks and a perceived greater freedom in not being forced to comply with the regulations of the community. This suggests that there is a scale that certainly consists of two opposites: conventional and the Slow Flower Movement, but there are also growers operating between these two poles. This could be interpreted as an ongoing process among field grown cut-flower growers in developing the niche and future direction(s). Evidently, some growers are exploring how to structure a position and foundation, which enables them to balance between what they perceive as not becoming too similar to conventional production but at the same time, possibly, not becoming too rigid.

From the growers’ perspective, it is the applied cultivation techniques, i.e. the craftsmanship, the ban on pesticides, mineral fertilisers as well as the lack of long-distance transports, which make up the major cornerstones of the product (cut-flowers) and subsequently, the value they deliver to their customers.

*Yes, so they get sustainably and environmentally friendly grown flowers and like locally produced and all that means, when you appreciate it of course. And then you get the experience of walking around and pick your own flowers… (R3)*

This production system, thus, partly testifies what can be described as a return to old technologies, mainly based on manual work and an active stance against the use of machines. This provides a good example of the link to the slow movement and the conviction that fast (machines) is bad and slow (manual work) is good. The historical connection is also partly visible in the cultivated assortment, for example, Aster, Dahlia, Phlox, Delphinium and Gillyflower, which were common in production during the 1930s (Berge, 1939). The varieties of the cultivars produced within this system, compared to conventional floriculture are often selected in line with a strategy of differentiation. One example is Valentine's Day, a day traditionally associated with roses. However, due to climatic reasons it is not possible to provide domestically produced roses in February. Instead, domestic growers market e.g. *Helleborus*, dried flowers and *Salix*. The assortment is, hence, focused on uniqueness, e.g. through growing different varieties, but also through a quality described as wilderness and imperfection, as compared to what is perceived as perfection in conventional cut-flowers. To address this, uniqueness and specific popular varieties are central when communicating with customers through social media, such as Instagram. With this, the growers add a value-adding factor that distinguishes and differentiates the cut-flower field growers' businesses compared to conventional import produce (Pölling et al., 2016). The result, thus, supports the findings presented by Short et al. (2017), who described how cut-flower field growers compete with the conventional flower business by focusing on exclusiveness and uniqueness. The findings also illustrate what Grönroos (2008) explains as adding value without changing the use of the product, and how this can support firms to distinguish their business compared to the competitors.

Sales channels are implemented through a broad diversity of added value options (Lu and Dudensing, 2015), such as self-picking (self-service or by guidance from the grower), flower trolleys with ready-to-go bouquets, stores and local farm shops, short food supply chains and home delivery. In addition, growers also sell to other firms, such as event planners, restaurants and florists. The short sales channels enable the growers to cut out several intermediaries, which is seen as advantageous in improving financial margins and revenues, in line with findings presented by Lu and Dudensing (2015). According to the growers, selling directly to consumers is the most profitable sales option. The growers often apply flexible and generous opening hours, e.g. opening hours during afternoons, evenings and weekends. Customers buy flowers for several reasons as well as specific events: gifts, weddings, funerals, christenings and/or decoration. Growers are most aware that their business attracts customers from a large geographical spread, local as well as more distant, regular customers as well as new ones, which is beneficial for the grower but also for the surrounding companies in the local area. The growers explain that customer demand has historically exceeded the available supply, which has forced them to turn down requests from both florists and private consumers. However, an experienced lack of production capacity has not necessarily led to growers expanding their production; instead, it has implied a caution among growers in initiating new collaborations with resellers and florists.

Since a majority of all work at the production site is done by hand, e.g. preparing flower beds, watering, dead-heading, weeding and tying bouquets, muscle strength is a very important resource. However, even though the movement has a strong focus on avoiding novel technology in the production, some growers have adopted the use of various technical equipment in the production system, such as underfloor heating, LED-lamps, vertical shelf systems, green houses, tunnels and at times drip irrigation. Not having access to a green house or a tunnel was perceived as a most limiting factor, and several growers use tunnels to extend the season to store or protect plants. Organic soil, natural fertilisers (manure from horses, cows and hens, compost, and grass from mowing the lawn and seaweed), trays, pots and tools constitute the necessary production material. The availability of seeds, and especially seeds that reach specific qualities, such as e.g. unique varieties and a broad diversity of varieties, are regarded as essential to the business. The need for foreign seeds clarifies the high dependency on foreign established horticultural seed companies and international transportation, due to the lack of domestic alternatives. Brexit has, however, made it more difficult to buy seeds from high-quality seed-companies in England, which is perceived as an aggravating circumstance.

*I have actually ordered mainly from England until now; then, you will see later. Now I have pretty good contacts in England, so I can probably get help with sending them here, so I think I will continue to do so because the varieties I grow, cannot really get them here and the quality... (R4)*

Finally, all four growers own their land, which is regarded as an essential asset. Access to water is also seen as vital for production and having a private well instead of municipal water is advantageous, mainly due to water restrictions during summer. Costs were highest during the start-up phase, mainly due to investments in supplies, such as cultivation pots, LED lights, and other supplies necessary for the production. Time (labour-costs) was perceived as the most important and limiting of all costs. Currently, none of the growers can take out a salary for all the time they spend in the cultivation. Moreover, some growers have either full-time or part-time employment, supplementing the income from the cultivation. Identified struggles with increased costs, risk in production and not the least the complexity in the development, are congruent with the struggles identified by Harvardi-Burger et al. (2020) when implementing sustainable innovations.

Customer relationship is most central to the economic layer of the business model, and the growers emphasise the importance of being present and available when customers are at the flower farm. Growers expect their customers to be interested, knowledgeable and willing to pay for unique varieties of flowers, which is in line with the perceived importance of interaction, guidance and/or providing consumers with help if needed. Even though sales channels generally are dominated by direct sales, two of the interviewed growers mainly sell to florists. This is surprising since the growers report better profitability when selling to private persons. Reasons for the chosen strategy could be the need to have additional jobs on the side, but also convenience in having only one customer, compared to several private customers. This may also be interpreted as contributing to the differentiation and development of the emerging niche of slow flower and making these flowers available to urban customers not located close to the farm or having access to/interest in using a car to reach the production site.

The use of social media was identified as a most important communication part of the businesses and of the available options, Instagram was the most frequently used service. According to the growers, their use of social media was mainly implemented with the aim of increasing sales. Yet, none of them claimed to have a strategy for their use of social media, which Gholston et al. (2016) describe as typical of how small businesses use social media, even though such an approach does not always collaborate very well with the customers. Instead, Zhu and Chen (2015) describe how the user's (e.g. customers) motives for using social media mainly represent a means to socialise and connect with other people. However, despite the lack of a formalised strategy, all growers prioritise a good customer relationship, which mainly relies on activities on social media (e.g. Instagram and Facebook), e-mail, or through their web page.

*I know that some are very skilled, but I'm not looking to get as many followers as possible. That's not what drives me. Instead, I want to reach the people in the area who buy my bouquets. But of course, you can have a clearer profile, but as I said, well I don't know... (R2)*

This strategy additionally highlights the importance of the interaction that takes place between the customers and producers and how this relationship constitutes the core of the applied marketing. The result exemplifies how social media (e.g. Facebook, Instagram) nowadays function as an important component of the studied businesses' environments and represent an easy-to-use marketing tool that allows businesses, such as these, to quickly and cost-effectively reach out to and engage with their customers and build communities (Devereux et al., 2019). The importance of applying this strategy is further explained by Harvard (2019) who describes how social media users, in combination with social structures, often experience themselves as being close to or knowing the person (or company) behind a specific account, even though they have never even met. By following these accounts on social media, the followers can get access to what they interpret as real insights into the account owner's life or business. The identified use of relationship marketing and how the owners use co-creation of value to build and enhance customer relationship (Payne and Frow, 2017) is, thus, identified as one of the most important actions that these firms engage in. The successful use of social media suggests that these owners have managed to use their social media platforms efficiently as well as understand what draws attention and interest from their customers.

Since the majority of firms are located in rural areas, growers regard their social media platforms as the company's display window; consequently, social media platforms are used for marketing and making the business visible, taking orders, inspiration, and competence development and finally, functions as a sales channel. Activities in line with how Gholston et al. (2016) illustrate how social media marketing constitutes an essential part of small businesses. However, despite the knowledge and the understanding that a more strategic marketing approach most probably would have been beneficial, such actions were not taken by the growers, mainly due to time constraints.

### 4.3 The environmental layer

Growers mainly perceive that the environmental production is linked to the craftsmanship and manual work (avoidance of machines and the use of fossil fuels); use of organic soil; ban of pesticides as well as mineral fertilisers; avoiding the use of greenhouses; local, short distribution chains; and contributing to biodiversity (a wide variety of flowers, favourable for bees and insects). The environmental impact of the production is of concern to the growers; however, no attempts had been done to produce measurable objectives, such as Life Cycle Assessment, to concretise the environmental impact of their production unit, and/or to communicate and explain which ecosystem services the cultivation contributes to. The production itself and the methods it is based on are considered sufficient; furthermore, developing a method of measuring, formalising and jointly communicating embedded environmental values is perceived as unnecessary.

Some of the growers started their business mainly due to environmental concern, whereas others have become more interested and knowledgeable in environmental issues, as part of the process of developing their firm.

*…that you grow sustainably, no toxins, just natural fertilisers; it is also about the biological diversity. Because when you grow so many kinds of flowers and also those that bees and such like, it will be in harmony with nature and animals as well and it is important. I dry flowers to use for other events, and I only grow in the open without heating; that's also part of it. (R4)*

The flowers are mainly produced and distributed locally, which the grower believes to be of environmental benefit. It is, however, worth noting that seeds and bulbs are imported, and that the production relies on input of additional production material (soil, pots, etc.). The direct sale from the farm or through local shops or alternative points of sale is also based on customers using means of transport, such as by foot, bike, car or, when available, public transport, to reach the cultivation. Such means of transportation are considered more environmentally friendly compared to importing flowers and the frequent use of air-born transport within the conventional floriculture production system. Growers also address how their work and interactions with consumers contribute to an increased public awareness of the environmental advantages of choosing domestically produced cut-flowers, instead of imported.

Growers additionally express that the flowers per se are compostable and that they, for example, use compostable thread in their bouquets. This illustrates the conscious choices made, with the aim of reducing waste and supporting circularity through keeping flowers and added material compostable. The fact that the flowers are harvested at the time of purchase, or just shortly before, is considered to contribute to better durability (vase life), compared to conventional products. According to the growers, customers express a great appreciation for the product's long-lasting vase life, which is estimated to last up to two weeks, depending on the specific cultivars and plant development phase.

*…but all at least one week, they do. It's clear that it depends on what kind of variety it is, but if you say a week at least on average, then sometimes people come back and just – Oh I picked them and they've been there for two weeks now and they are really nice. But that depends on the variety and such, of course. (R3)*

The growers also explain how they provide guidance during the self-picking on which cultivars to choose when requesting a long-lasting bouquet. Some growers determine their selection of cultivars on the criterion that the flowers should have a long vase life.

The Nordic climate and the requirement that all cultivation must be in open field and without the use of protective environments, such as greenhouses, hinders continuous production all year around. Growers support this position (that import should be avoided), suggesting a ban of imports when domestic production is shut down due to seasonal circumstances. Instead, everlastings and other dried flowers are suggested to be used when climate conditions prevent production of domestic fresh flowers.

### 4.4 The social layer

Growers mainly work alone in their business and regard themselves as self-employed, one-woman, entrepreneurs. With that being said, they also strongly emphasise their dependency on resources such as unpaid work contribution from family members. Growers’ working conditions often involve very long working days during high season and stress due to handling both production and external communication (marketing, updating social media). As the cultivation is often in close proximity to the private home, it is also difficult to have clear boundaries between work and leisure and to clearly show demarcations between private and commercial parts of the garden. By offering customers flexible opening hours, customers may believe that it is always open, which may lead to anxiety, irritation and misunderstandings.

The perceived aesthetic value and the emotional qualities of the physical place and its surroundings, as well as the experience of the cut-flowers per se, are of high importance to the growers. These values are often referred to as “*the beauty of it*”, which is interpreted as an overall positive quality of the land, the closeness to plants (interaction and work conducted in the field), the applied landscape design and the products (the flowers). These positive values were often of central significance to why growers had chosen to start their business as well as why they continued, despite difficulties with profitability and lack of time.

The growers also perceive themselves as ambassadors for an entrepreneurial rural lifestyle. Some growers are part of local (female) entrepreneurial networks. Additionally, they express an awareness of their value in local business clusters and believe that their activities may attract new actors and firms, which, in turn, may lead to a more flourishing countryside. Growers are, thus, most aware of and proud to contribute to the local economy to which they belong.

Moreover, the growers are careful to point out that their production units not only add beauty to people residing in their garden or fields, but that it is also of benefit to neighbours as well as people passing by. One grower describes how she perceives the customers' reaction to the environment on her flower farm and her own feelings of going out in the cut-flower field gardens with enjoyment:

*That they come out in a quiet place in nature, many say; oh god, how beautiful it is here and oh, how nice and quiet and calm it is. You feel like how they relax and are completely taken by the place. We live here, like every day when I go out, I also feel that it is such a heavenly quiet and beautiful place where you can really breathe out, no stressful cars, no noise and so on. (R1)*

The result illustrates a perceived positive value of residing in the cultivation, both among growers and their customers. Growers also express the experience of a significant social value that arises from the personal meeting between them and their customers, both in person, but also through social media and e-mail. In addition, the location also acts as a place for socialising and dialogue among customers. Especially when customers are given the opportunity to sit down and rest and/or have coffee or provided space for a picnic. The social value of the close connection between the growers and their customers enables the growers to not only provide knowledge but also interact. Following this, growers and customers co-create value through providing knowledge of techniques and aesthetic aspects to consider when customers produce their own bouquets. This finding is interesting, as it provides an example of the Service-Dominant logic and the understanding of how a marketing and purchasing landscape can be built on reciprocal communication (Lusch and Vargo, 2006) as well as how value propositions are reciprocal and jointly created, linking the firm and its customers (Ballantyne et al., 2011; Grönroos and Voima, 2013). Growers also describe that their self-picking could be seen as an event and social gathering which contributed to the local culture and society. Regarding the fact that the activity is mainly conducted outdoors, it was most appropriate during the restrictions due to the Covid-19 situation in 2020 and the need for people to identify outdoor activities for social purposes.

## 5 Conclusions

The emergence of the Slow Flower Movement indicates a consumer demand for a sustainable alternative to the conventional cut-flower industry. This study highlights how growers within this movement have developed their business models to provide products that weave together several values linked to the sustainability of cultivating field grown cut-flowers. Flowers produced in line with the Slow Flower Movement do provide unique sustainable values, such as contributing to biodiversity; a different assortment and function as a meeting point; and contribute to the local culture, landscape and society. Growers report a stable and increasing consumer demand, indicating a growing market for domestic production of cut-flowers. However, despite the growing market, the result also points to economic and social difficulties in these firms, mainly linked to profitability and work conditions. When comparing to conventional firms, the production within the movement is done at a much smaller scale, which partly may explain identified difficulties to reach profitability. To deal with these circumstances, several of the interviewed growers run additional side businesses to complement their income, resulting in diversity in applied business models and firm strategies. The strong link to the Slow Movement is dominant and is especially exemplified in the great emphasis on time consuming manual work, despite the heavy workload this implies and how this probably negatively affects profitability. A conclusion is also that the main target group for these products appear to be a more privileged consumer segment, which raises questions if these products are for people in general, in line with the concern put forward by Vostel (2019) (in relation to the Slow Food Movement). A lack of statistics and data on production cost, sales volumes, price and environmental impact within the Slow Flower Movement system complicates an overview of the scope and size of the production, as much as it hampers a comparison regarding e.g. productivity and environmental benefits, compared to the conventional production system.

The deeply embedded culture of high social media activity among the growers is identified as one of the most important key activities contributing to and driving the increase, not only in demand but also attracting new growers. Social media platforms, thus, represent a crucial tool in the development of the Slow Flower community and the movement surrounding Swedish cut-flower production and should be seen as a central foundation for its establishment.

As previously stated, profitability is of major concern for several growers. Identified solutions include, for example, scaling up production and hiring staff, or scaling down the production and focusing on one specific target group. Additional solutions could also be to adjust pricing and apply a pricing strategy, building on differentiation, addressing the uniqueness of the flowers and communicating the values that are embedded within the slow flower system, compared to the conventional floriculture. Given the identified power in using social media, developing skills in implementing and using such tools additionally indicates an opportunity for less experienced growers to increase profitability.

Finally, it is interesting to notice that the movement heavily relies on the conviction that work conducted in the cultivation should preferably be done by hand and with old methods. However, there is no discussion putting forward that the marketing of the products should be done by using old methods and by avoiding the use of technical innovations, such as social media. Evidently, such a standpoint would have been of great detriment to the growers. The lack of discussion on this fact is interesting and may point to a need to explore why some technologies are perceived as relevant and support the authenticity of the production, whereas others are not. It also clearly illustrates an example where an innovative technology has been accepted and implemented in the movement. Future studies should explore why the openness to the technology underpinning social media is uncriticised, compared to the adoption of production technology. It should, however, be added that some of the decisions (e.g. avoiding the use of greenhouses) primarily originate from sustainability arguments. Presently, growers are allowed to use tunnels; however, by using greenhouses, the season for domestic cut-flowers would be even more prolonged. Considering the central part that flowers have in human culture and linked economic, environmental and social benefits, it is of importance to continue to scrutinise the sustainability, not only in the Slow Flower Movement but also explore future links to knowledge and technical innovations applied in conventional cut-flower production. By following such an approach, these presently opposite movements could hopefully be of mutual benefit to each other in the development of future, domestic, sustainable production of cut-flowers.

It is important to highlight and keep in mind that this study only examines the Slow Flower Movement in Sweden, limiting the selection of participants to Swedish cut-flower field growers. Even though resembling increasing popularity of local field-grown cut-flowers is observed in several other countries, the results of this study should not be directly transferred to other contexts as there may be country-specific differences, in, for example, consumer demand, cut-flower supply system and cultivation opportunities. Considering the low number of studies on the topic of the Slow Flower Movement, a need for additional studies is suggested. We also believe that future studies should compare applied business models and value delivered within the domestic cut-flower industry with other small-scale firms operating within horticulture and agriculture. Due to the identified difficulties in achieving profitability, it is of great importance to identify transferable success factors for the development of small-scale firms. Finally, the understanding that a majority of the Swedish cut-flower field growers are female calls for addressing the gender perspective and exploring possible significance and implications of this fact.

## Declarations

### Author contribution statement

Rebecca Thörning: Conceived and designed the experiments; Performed the experiments; Analyzed and interpreted the data; Wrote the paper.

Åsa Klintborg Ahlklo: Conceived and designed the experiments; Analyzed and interpreted the data; Wrote the paper.

Sara Spendrup: Conceived and designed the experiments; Analyzed and interpreted the data; Wrote the paper.

### Funding statement

This research did not receive any specific grant from funding agencies in the public, commercial, or not-for-profit sectors.

### Data availability statement

The data that has been used is confidential.

### Declaration of interest’s statement

The authors declare no conflict of interest.

### Additional information

No additional information is available for this paper.
